# Urinary Bisphenol A Levels in Girls with Idiopathic Central Precocious Puberty

**DOI:** 10.4274/Jcrpe.1220

**Published:** 2014-03-05

**Authors:** Erdem Durmaz, Ali Aşçı, Pınar Erkekoğlu, Sema Akçurin, Belma Koçer Gümüşel, İffet Bircan

**Affiliations:** 1 Mersin State Hospital, Department of Pediatric Endocrinology, Mersin, Turkey; 2 Hacettepe University Faculty of Medicine, Department of Toxicology, Ankara, Turkey; 3 Akdeniz University Faculty of Medicine, Department of Pediatric Endocrinology, Antalya, Turkey

**Keywords:** Bisphenol A, endocrine disruptor, idiopathic central precocious puberty, sex hormones

## Abstract

**Ob­jec­ti­ve**: Bisphenol A (BPA) is an industrial chemical, particularly used to harden plastics. BPA is thought to have negative health effects on both laboratory animals and humans. Consider ing the decline in age of onset of puberty noted in recent years, particularly among girls, the importance of BPA as an estrogenic endocrine disruptor has increased. In this study, we aimed to determine urinary BPA levels in girls with idiopathic central precocious puberty (ICPP).

**Methods**: Non-obese girls newly diagnosed with ICPP (n=28, age 4-8 years) constituted the study group. The control group consisted of 25 healthy age-matched girls with no history of ICPP or any other endocrine disorder. Urinary BPA levels were measured by using high-performance liquid chromatography.

**Results**: In the ICPP group, urinary BPA levels were significantly higher compared to the control group [median 8.34 (0.84-67.35) μg/g creatinine and 1.62 (0.3-25.79) μg/g creatinine, respectively (OR=8.68, 95% CI:2.03-32.72, p=0.001)]. There was no marked correlation between urinary BPA levels and body mass index in either group. In the ICPP group, no significant correlations were found between urinary BPA levels and serum luteinizing hormone, follicle-stimulating hormone and estradiol levels.

**Conclusions**: To our knowledge, this is the first study evaluating the urinary BPA levels in Turkish girls with ICPP. Our results indicate that the estrogenic effects of BPA may be an etiologic factor in ICPP.

## INTRODUCTION

Precocious puberty (PP) is defined as the beginning of secondary sexual characteristics before age 8 years in girls and 9 years in boys (1). It is usually caused by premature activation of the hypothalamic gonadotropin-releasing hormone (GnRH). This condition is described as central PP (CPP) (2). Only ∼20% of girls with PP have an organic etiology (including central nervous system lesions or congenital adrenal hyperplasia) (2). In most cases, no underlying etiology of PP can be identified and the condition is labelled as idiopathic CPP (ICPP).

A decline in age of onset of puberty has been observed particularly among girls in the US in the mid-1990s (3,4). In a recent paper, Biro et al (5) reported that the number of girls entering puberty at early ages in the US showed a marked increase in recent years. The researchers reported that about 16% of girls entered puberty by the age of 7 and about 30% by the age of 8. This phenomenon is now also encountered in Europe (3,6). Several factors, like genetic predisposition, psychosocial and socio-economic conditions, nutrition, ethnicity and increased prevalence of adiposity may contribute to this phenomenon. The widespread presence of endocrine-disrupting chemicals (EDCs), in particular estrogen-like EDCs in the environment, is also suspected to contribute to the trend of early pubertal onset (3).

Estrogen-like EDCs are exogenous, man-made chemicals which alter the functions of the endocrine system. Bisphenol A (BPA) is considered as an estrogen-like EDC and it is one of the highly abundant chemicals worldwide (7). BPA is formed as an intermediate compound in the manufacturing process for polymers, epoxy resins, flame-retardants, rubber chemicals and is available in reusable baby and water bottles and in the inner lining of food and beverage cans (8).

Although the mechanism is not still clear, there are some reports that perinatal exposure to BPA is associated with early pubertal onset in female rats and mice (8,9,10,11). BPA functions as a xenoestrogen. It binds to estrogen receptor α (ERα), ERβ and strongly to ERγ (12,13,14). In addition, BPA may cause weight gain which is frequently associated with reproductive dysfunction and early onset of puberty both in animals and in humans (15,16).

Urinary concentrations of total (free plus conjugated) BPA, particularly in spot samples, have often been used to evaluate exposure to BPA from both environment and diet. Available data from biomonitoring studies in different continents, suggest that human exposure to BPA is widespread across the lifespan in these parts of the world. Biomonitoring-based median exposure estimates are in the range of 0.01-0.05 μg/kg body weight (bw) per day for adults and higher (0.02-0.12 μg/kg bw per day) for children. The 95th percentile exposure estimates are 0.27 μg/kg bw per day for the general population and higher for infants (0.45-1.61 μg/kg bw per day) and children 3-5 years of age (0.78 μg/kg bw per day). These estimates are comparable to those based on concentrations in food and amounts of food consumed. BPA has a relatively short elimination half-life (<2 hours for urinary excretion) (17).

To our knowledge, there are no reported studies investigating the relationship between urinary BPA levels and ICPP. Based on this background and taking into account the frequency of high BPA exposure in humans, this study was designed to investigate the urinary BPA levels in Turkish girls with ICPP.

## METHODS

The subjects recruited in the study consisted of girls aged between 4 and 8 years and were grouped as follows:

The control group comprised 25 healthy girls with no history of ICPP. Girls with any endocrine disorder and those who showed any secondary sexual characteristic were excluded. Control subjects were assessed for a second time one year later for evaluating any sign of pubertal development. At their second visit, girls showing pubertal signs, such as premature thelarche (PT) and premature pubarche, were excluded from the study.

The study group consisted of 28 girls with ICPP. The diagnosis of ICPP was made in accordance with the following criteria: (i) breast budding appearing before age 8 years, (ii) advanced bone age >1 year above chronological age and (iii) luteinizing hormone (LH) peak values >5 mIU/mL at GnRH stimulation test (Gonadorelin acetate, Ferring®) (18).

Girls younger than four years were not recruited in the study because of difficulty to obtain the urine samples and risk of contamination of the special bottles.

All subjects were born and were still living in the Antalya province of Turkey and were examined by the same pediatrician. Weight status was determined as body mass index (BMI) and obesity was defined as BMI >95th percentile according to national standards (19). Children with a diagnosis of CPP, a history of exogenous exposure to estrogen-containing pills or creams, benign non-progressive PT and those with a family history of PP were not included in the study. Obese children were also excluded.

All ICPP subjects underwent cranial magnetic resonance imaging (MRI) in order to exclude intracranial pathologies. Plasma basal LH, follicle-stimulating hormone (FSH) and estradiol (E2) levels were also measured in the first visit of ICPP group.

Spot urine samples from both the study and control groups were collected into deplasticized glass beakers between September 2010 and February 2012 in the Pediatric Endocrinology outpatient clinic. The samples were aliquoted and kept at -20 OC. The urine samples of all subjects were sent to Hacettepe University, Faculty of Pharmacy, Department of Toxicology in dry ice. All samples were kept at -20 OC Cuntil the extraction of BPA.

All subjects participated in the study voluntarily and a written consent was obtained from the parents of the children recruited in the study. The study was approved by Akdeniz University’s Ethics Committee.

**Deplasticization of the Glassware**

Extreme caution was taken in order to prevent contact with plastic material throughout the study. All the glassware used for the collection of urine samples were deplasticized with tetrahydrofuran: n-hexan (50:50, v/v) for 2 h and later were kept in an incubator for 2 h. All the test tubes were deplasticized on a heater at 400 OC for 4 h.

**Chemicals**

All the chemicals including BPA were obtained from Sigma-Aldrich (St. Louis, MO). Glucuronidase/arylsulfatase enzyme (from Helix pomatia) was purchased from Roche (Mannheim, Germany). All high-performance liquid chromatography (HPLC) equipment were obtained from Agilent (Santa Clara, CA). LH, FSH and E2 electrochemiluminescence immunoassay kits were from Roche (Mannheim, Germany).

**Extraction of BPA from Urine**

For the analysis of urinary BPA, the method of Yang et al (20) was used with some modifications. Briefly, after spiking urine (500 μL) with BPA (5 ng/mL), sodium acetate buffer (200 M, pH 5) and glucuronidase/arylsulfatase were added and the mixture was incubated at 37 OC for 3 h in order to free the conjugated BPA. After incubation, HCl (2 N, 100 μL) and ethyl acetate (5 mL) were added, centrifuged and the supernatant (2.5 mL) was evaporated; the glass tubes with residues were kept at -20 OC until analysis.

**Chromatographic Analysis**

The residue was dissolved in acetonitrile (60%, 400 μL) and 100 μL of the sample was injected to HPLC (Hewlett-Packard Agilent 1100 series, Santa Clara, CA). HPLC parameters were as follows: BPA standards used were 1.25, 2.5, 5, 10, 25, 50 and 100 ng/mL; C18 column (25 cm x 5 μm x 4.6 mm i.d.); column temperature: 25 OC; fluorescence detector (λexitation=230 nm, λemission=315 nm). Mobile phase was acetonitrile: tetrahydrofuran (2.5%) (gradient elution was applied as 60:40- 5:95), flow rate was 0.4 mL/min; retention time was 18.3-19.2 minutes and analysis duration was 40 minutes. The limit of detection (LOD) according to the method of the U.S. Environmental Protection Agency (EPA) for BPA was 0.5 ng/mL and the limit of quantitation (LOQ) was 1.25 ng/mL (21). The urinary BPA levels were calculated by the peak heights obtained from the chromatogram. Recovery studies were performed on urine samples spiked with 5 ng/mL of BPA. The average recoveries were found to be (mean±SD) 97.37±1.23% on ten occasions. Between-run precision was 2.76±0.24% coefficient of variation (CV) and within-day precision was 2.63±1.23% CV.

Urinary creatinine concentrations were analyzed simultaneously by HPLC according to Jen et al (22) with slight modifications and the urinary BPA concentrations were adjusted by urinary creatinine concentrations.

**Statistical Analysis**

Statistical analysis was conducted using PASW (Predictive Analytics SoftWare) Statistics, Release, Version 18.0.0 (SPSS, Inc., 2009, Chicago, IL). The distribution of BPA values was analyzed by using the Shapiro-Wilk test. The comparison between 2 parametric values was performed by using student’s t-test and for nonparametric values; Mann-Whitney U-test was used. The correlations of urinary BPA levels with BMI, LH, FSH and E2 values were assessed by using the Spearman’s rho (ρ) correlation test. A p-value of <0.05 was accepted as statistically significant.

## RESULTS

Two of the subjects from the ICPP group and 3 from the control group were excluded from the analysis because their values were below the detection limit. One subject from the control group did not show up for the first year follow-up. At the initial evaluation, nearly 85% of the ICPP group (n=22) had already reached Tanner 2 stage breast and pubic hair. Therefore, no statistical correlation could be determined between BPA levels and pubertal stages.

The clinical characteristics of the subjects with ICPP and the control group are shown in Table 1. There was no statistically significant difference between the two groups.

**Figure 1:**
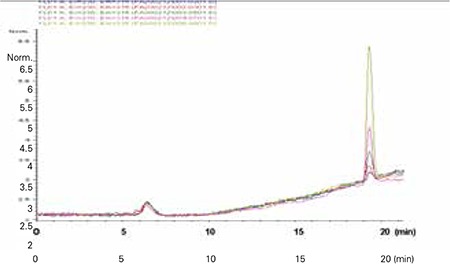
High-performance liquid chromatography (HPLC) chromatogram of bisphenol A (BPA) standards. Calculations were made according to the peak heights. Retention times were between 18.3-19.2 minutes

**Figure 2:**
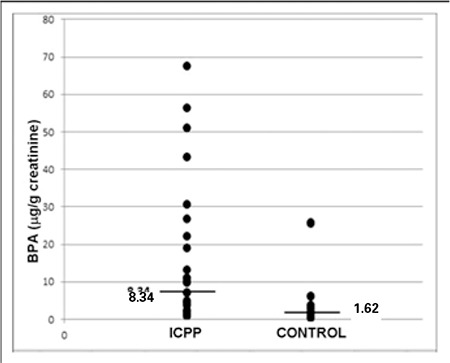
The distribution of urinary bisphenol A (BPA) levels in idiopathic central precocious puberty (ICPP) subjects and controls

An HPLC chromatogram for BPA is given in Figure 1. Urinary BPA levels were significantly higher in the ICPP group (n=26) compared with the control group (n=21) [median 8.34 (0.84-67.35) μg/g creatinine and 1.62 (0.3-25.79) μg/g creatinine, respectively (OR=8.68, 95% confidence interval: 2.03-32.72, p=0.001)] (Figure 2).

Urinary BPA levels did not correlate with basal FSH, LH and E2 levels in the ICPP group.

## DISCUSSION

In school-aged girls, accumulated data indicate a secular trend towards an increasing frequency of PP throughout the world (3). Available data on these topics are still very scarce, although the prevalence of PT and PP in different countries and the factors causing these conditions have become an issue of primary interest to pediatricians and endocrinologists in the last decade. It has been suggested that both PT and PP may be attributed to in utero or childhood exposure to estrogen-like compounds in the environment, particularly to estrogen-like EDCs (23). These synthetically produced compounds, including BPA, are ubiquitous in the environment and are present in many pesticides, plastics, baby products and other products used widely in daily life (24).

In this study, a significant difference (p=0.001) was observed in the urinary BPA levels of ICPP subjects and healthy controls, suggesting that BPA exposure may be associated with PP in girls. To our knowledge, this is the first study investigating the relationship between urinary BPA levels and ICPP in Turkish girls. Thus far, there are few epidemiological studies that have examined the relationship between BPA exposure and pubertal development and one study conducted to investigate the relationship between urinary BPA levels and PP in Chinese girls (25,26,27). 

Wolff et al (25) conducted a cross-sectional study examining the correlation between concurrent BPA exposure and pubertal development, breast development and pubic hair development in 192 American girls (mean age: 9.5±0.3; age range: 9.002-9.998). These authors reported that there was no significant difference between the urinary BPA levels of breast stage 1 and breast stage 2 groups nor between pubic hair stage 1 and pubic hair stage 2 groups. One year later, a large multi-center prospective cohort study performed on 1151 American girls (age: 6-8-years) was published. This study evaluated the relationship between urinary BPA concentrations and pubertal development (26). No statistical correlation was found between BPA levels and early pubertal development. Moreover, there were both overweight and obese girls in the mentioned study (32% of the girls were above the 85th percentile and 17% of the girls were above the 95th percentile) and interpreting the results seem to be more unfeasible. The authors did not report whether BMI was a modifying factor in pubertal development or whether BMI was correlated to urinary BPA levels (26). As obesity is a risk factor in the development of PP, we included only non-obese ICPP subjects in the current study. Kaplowitz (27) reviewed the available data on the relationship between body fat and the timing of puberty.

Qiao et al (28) examined the correlation between serum BPA levels and PP. BPA was detected in the serum of 40.9% of PP girls (n=110) and only in 2% of the control girls (n=100). Serum BPA levels of Chinese PP subjects showed a positive correlation with uterus and ovary volumes. The limitation of this study was the low number of control samples detected to be contaminated with BPA. Moreover, BPA concentrations in blood decrease quickly after exposure and are considerably lower than those in urine (17). Therefore, it is a better strategy to measure urinary BPA, rather than blood BPA levels. Calafat et al (29) reported that 93% of the American general population of ages 6 years and older had detectable urinary BPA levels. In the present study, 93% of ICPP and 88% of control group had detectable urinary BPA levels, a finding in line with the mentioned study.

Although the current study was conducted on a relatively small number of subjects, we found a significant difference (p=0.001) in urinary BPA levels between the groups. The BPA concentrations, particularly those of the control group [1.62 (0.3-25.79) μg/g creatinine], were comparable with those reported in studies performed in the US (26,27). It was reported that median urinary BPA concentrations in 1151 American girls (age: 6-8 years) ranged between 1-8.7 μg/g creatinine (26). In a study performed by National Health and Nutrition Examination Survey, the urinary BPA levels of ∼1500 children (300-400 children for each year) were measured in different years. In 2003-2004, the geometric mean was 4.32 μg/g creatinine (3.63-5.14), while in 2005-2006, it was 3.14 (2.79-3.54) μg/g creatinine, in 2007-2008, it was 3.05 (2.73-3.41) μg/g creatinine and finally in 2009-2010, it was found to be 2.36 (2.09-2.65) μg/g creatinine (30).

With our study design, it is not possible to explain the mechanism underlying the relationship between urinary BPA levels and ICPP. Kisspeptins play a crucial role in the timing of puberty and there are some reports that BPA altered the hypothalamic expression of Kiss1/kisspeptin in rats and mice (31). However, BPA may also induce ICPP by a different mechanism other than kisspeptin signaling (8). Patisaul et al (32) investigated BPA exposure in ovariectomized adult female rats and found no impairment in the signaling from kisspeptin neurons (from anteroventral periventricular (AVPV) to GnRH neurons). Moreover, studies performed on female rats showed that following developmental exposure to BPA, puberty was advanced; however, neither post-pubertal GnRH activation was impaired, nor AVPV or arcuate kisspeptin fiber density was changed (10,31). Recently, Kwon et al (33) examined serum kisspeptin and BPA levels of girls with CPP (n=31) and prepubertal age-matched healthy controls (n=30). Serum kisspeptin levels were significantly higher in the CPP group compared with the control group; however, BPA levels had no influence on kisspeptin levels. The researchers concluded that BPA may induce CPP by a different mechanism other than affecting kisspeptin signaling.

In a study evaluating the effect of BPA on estrogen receptor (ER) expression during the peripubertal period in the hypothalamus of male and female rats, the researchers determined that in females, BPA exposure increased the immunoreactivity of ER in the ventromedial hypothalamus, while in males, no increase was observed in the ER immunoreactivity in any of these regions (34). It might be postulated that disrupted signaling through ERs might be a mechanism by which BPA exerts neuroendocrine effects (8). BPA may also modulate LH and FSH levels (35). In male rats, postnatal exposure to high levels of BPA led to an elevation in serum FSH levels during the peripubertal period and to a decrease in both serum E2 and testosterone levels (36,37). In the current study, we did not observe any correlation of urinary BPA levels with serum LH, FSH and E2 levels which were measured at the first visit of the subjects. However, as we did not measure the sex hormone levels of the control group, making a comparison between the two study groups was not possible.

There are many reports about the decline in age of onset of puberty, particularly among girls, all around the world, including Turkey (38). Although our study has some limitations due to the smallness of the sample size, this is the first study determining the relationship between urinary BPA levels and ICPP in non-obese Turkish girls. In conclusion, we can postulate that estrogen-like EDCs like BPA might be one of the underlying risk factors for early pubertal development. Being a common problem, this subject requires additional studies in larger case series to confirm our results and explain the cause-effect relationship.

**Acknowledgement**

This study was funded by Akdeniz University Scientific Research Unit grant 2010.04.0103.26

## Figures and Tables

**Table 1:**
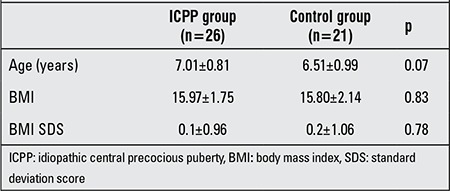
Clinical characteristics of the subjects in the study and control groups
